# Tight junction suppression by candidalysin promotes gut-to-blood dissemination of *Candida albicans*

**DOI:** 10.3389/fmicb.2026.1919478

**Published:** 2026-09-25

**Authors:** Yahui Feng, Wenjie Fang, Ning Zhang, Yiyang Wen, Dongmei Li, Dongmei Shi

**Affiliations:** 1 Department of Dermatology, Jining No.1 People’s Hospital, Jining, Shandong, China; 2 Laboratory of Medical Mycology, Jining No.1 People’s Hospital, Jining, Shandong, China; 3 Medical Aesthetics Department/Skin Health Management and Aesthetics Center, Jining No.1 People’s Hospital, Jining, Shandong, China; 4 Department of Dermatology, Shanghai Changzheng Hospital, Shanghai, China; 5 Department of Pathology, Jining No.1 People’s Hospital, Jining, Shandong, China; 6 Department of Microbiology and Immunology, Georgetown University Medical Center, Washington, DC, United States

**Keywords:** candidalysin, gut-to-blood, intestinal barrier dysfunction, paracellular invasion, tight junction disruption

## Abstract

**Background:**

The commensal fungus *Candida albicans*, a natural inhabitant of the human gut, can transition to a pathogenic state and disseminate into the blood. Disruption of the intestinal mucosal barrier, particularly through the impairment of epithelial cell integrity and intercellular junctions, is a critical step in this dissemination process. While candidalysin is a pore-forming cytolytic peptide encoded within ECE1 (extent of cell elongation 1 gene), its relationship to the abundance and organization of specific intestinal tight-junction proteins during dissemination remains incompletely defined.

**Methodology/principal findings:**

A murine model and human colonic epithelial cells (NCM-460) were used to investigate the effect of candidalysin on fungal dissemination and its association with changes in tight-junction protein abundance and distribution. *In vivo*, infection with a candidalysin-deficient *C. albicans* mutant preserved TJ architecture, substantially reduced pro-inflammatory cytokine responses, and substantially reduced the frequency and amount of detectable fungal DNA in blood. In contrast, infection caused by wild-type (WT) and *ECE1*-reconstituted strains resulted in elevated serum levels of the pro-inflammatory cytokines IL-6, IL-1β, and TNF-α, accompanied by reduced protein expressions of the TJ components (ZO-1, Occludin, and Claudin-1). *In vitro*, experiments using purified candidalysin demonstrated a biphasic, dose-dependent effect on TJ proteins. At 3.125 μg/mL, TJ-associated signals were preserved and selected protein measurements were modestly increased, whereas transcript levels showed a slight reduction. At higher concentrations (≥12.5 μg/mL, up to 50 μg/mL), higher candidalysin concentrations were associated with pronounced loss and discontinuity of TJ-associated signals and reduced abundance of selected TJ proteins.

**Conclusion/significance:**

The dose-dependent effects of candidalysin on epithelial junction-associated proteins support a context-dependent contribution to intestinal pathogenesis. These findings associate candidalysin with epithelial injury and dissemination in the present model but do not establish a binary molecular switch or a candidalysin-exclusive mechanism. Elucidating how candidalysin coordinates epithelial and immune pathways provides new insight into the mechanisms that sustain gut–fungal symbiosis and may reveal therapeutic targets to preserve mucosal integrity and prevent systemic candidiasis.

## 1 Introduction

Invasive candidiasis (IC) is a serious fungal infection predominantly caused by *Candida albicans*. It ranks among the leading causes of hospital-acquired fungal bloodstream infections and is associated with high morbidity, mortality, and significant therapeutic challenges. IC most often arises in individuals with impaired immunity (Chakraborty et al., 2025; McCort and Tsai, 2023; Wolfgruber et al., 2024). As a commensal resident of the human intestinal tract, *C. albicans* can undergo a morphological transition from a benign yeast form into invasive hyphae during infection. This transition, together with additional fungal virulence factors and complex interactions with host defenses, determines the progression and severity of disease (Chen et al., 2020; Phan et al., 2022).

Among the virulence factors of *Candida albicans*, the peptide toxin candidalysin has emerged as a key determinant of mucosal damage. Candidalysin is a hypha-associated fungal peptide toxin that is critical for mucosal infection (Moyes et al., 2016). It is a 31-amino-acid α-helical peptide derived from the ECE1 polyprotein and produced preferentially during hyphal growth (Moyes et al., 2016; Müller et al., 2024; Westman et al., 2022). The ECE1 gene was originally characterized in association with cell elongation in the dimorphic pathogen *Candida albicans* (Birse et al., 1993). As a pore-forming toxin, it polymerizes within host membranes, leading to membrane rupture, calcium influx, and host cell lysis. Beyond direct cytotoxicity, candidalysin also activates epithelial signaling pathways and promotes robust inflammatory responses, including the release of cytokine and formation of neutrophil extracellular trap (NET) (Lortal et al., 2025; Sprague et al., 2024). Through this dual action-direct epithelial damage coupled with immune activation-candidalysin impairs the mucosal barrier and facilitates fungal penetration into deeper tissues and the bloodstream (Liang et al., 2024; Song et al., 2022; Unger et al., 2023; Wu et al., 2023). Notably, while the above effects highlight candidalysin’s critical role in *C. albicans* pathogenesis, although candidalysin-dependent epithelial damage and intestinal translocation have been established, the contribution and regulation of specific intestinal tight-junction components *in vivo*, and their relationship to dissemination and residual disease severity, remain incompletely defined. Allert et al. (2018) demonstrated that candidalysin-dependent epithelial damage is a major determinant of *C. albicans* translocation across intestinal epithelial layers, while low-level candidalysin-independent translocation can still occur. More recently, Morelli and Queiroz (2025) used a three-dimensional gut-on-chip (OrganoPlate) model with TEER (transepithelial electrical resistance), protein detection, and immunostaining to show that candidalysin impairs epithelial barrier integrity and induces inflammatory responses. Building on these studies, the present work focuses on an *in vivo* isogenic comparison of wild-type, ece1-deficient, and ECE1-reconstituted strains, together with analysis of ZO-1, occludin, and claudin-1 at the protein and transcript levels in a human colonic epithelial-cell model. Specifically, the detailed mechanism by which candidalysin aids *C. albicans* in breaking through the mucosal barrier remains underexplored, leaving a key gap in our understanding of *C. albicans*’ invasive process.

Translocation of *C. albicans* from the intestinal lumen into the bloodstream represents a principal source of endogenous disseminated candidiasis, and candidalysin is also thought to further support *Candida* invasion by promoting nutrient acquisition from damaged tissues (Allert et al., 2018; Sprague et al., 2024). The gastrointestinal mucosa, as the body’s first line of defense, is therefore a critical site of candidalysin activity. Indeed, experimental interventions that protect or restore intestinal barrier integrity significantly reduce fungal burden in invasive candidiasis. Similarly, host-targeted strategies such as β-glucan supplementation, which upregulates tight-junction proteins and enhances epithelial integrity, have been reported to counteract candidalysin-associated inflammation and limit fungal invasion (Morelli and Queiroz, 2025; Swidergall et al., 2021). However, the specific link between candidalysin and the regulation of intestinal barrier components–particularly tight junction (TJ), which are central to barrier function–has not been systematically investigated.

Epithelial tight junctions (ETJs) play a critical role in maintaining intestinal barrier integrity and regulating selective permeability for nutrient absorption in the gut. Core components of intestinal ETJ protein include the proteins ZO-1, occludin, and claudin-1, whose expression levels are spatiotemporally regulated and directly influence barrier function (Alonso-Roman et al., 2022; Garg et al., 2025; Liu et al., 2021). Downregulation or mislocalization of these proteins leads to increased epithelial permeability and barrier disruption, thereby promoting inflammation and disease progression. Specifically, ZO-1 functions as a cytoplasmic adaptor protein that anchors transmembrane proteins such as occludin and claudin-1 to the actin cytoskeleton, ensuring the structural stability of tight junctions (Morelli and Queiroz, 2025; Sprague et al., 2024; Westman et al., 2022). Barrier impairment is therefore often characterized by both altered distribution and reduced expression of these key proteins (Bai et al., 2023; Huang et al., 2020; Liu et al., 2023; Kadry et al., 2021). Despite this, whether candidalysin disrupts the intestinal barrier by targeting ZO-1, occludin, or claudin-1–either by altering their expression or subcellular localization–remains a critical knowledge gap.

Addressing this gap is essential, as tight junction dysfunction is a pivotal event preceding epithelial barrier breakdown and systemic dissemination of *C. albicans*. In this study, we investigated the mechanisms by which candidalysin contributes to gut-to-blood dissemination, with particular attention to its dose-dependent effects on intestinal epithelial barrier integrity. In this study, we examined whether ECE1/candidalysin status is associated with intestinal TJ-protein abundance and distribution, fungal dissemination, and systemic inflammation in a controlled murine intraluminal infection model. We further characterized concentration-dependent TJ-associated responses to purified candidalysin in NCM-460 cells. Our aim was to define the relationship between candidalysin and specific TJ-associated phenotypes rather than to establish a complete signaling pathway, thereby compromising barrier integrity both *in vitro* and in an animal model. These results suggest that therapeutic strategies aimed at neutralizing candidalysin or reinforcing TJ protein expression may offer protective benefits against mucosal damage and invasive candidiasis.

## 2 Materials and methods

### 2.1 Cell line culture and strain source

The NCM-460 human immortalized intestinal epithelial cell line was purchased from Fenghui Biotechnology Co., Ltd. NCM-460 cells were maintained in high-glucose DMEM supplemented with 10% heat-inactivated fetal bovine serum and 1% penicillin–streptomycin at 37 °C in 5% CO_2_. For immunofluorescence, qPCR, and Western blot experiments, cells were transferred to serum-free medium 12 h before candidalysin exposure and maintained under serum-free conditions until harvest.

### 2.2 *C. albicans* strains and culture

All *C. albicans* strains used in this study were previously gifted (Moyes et al., 2016). The numerical identifiers 1477, 2057, and 2059 were assigned by the strain source/laboratory. For clarity, the strains are designated throughout as WT [1477 = WT (1477)], ece1Δ/Δ [2057 = ece1Δ/Δ (2057)], and ECE1-reconstituted [2059 = ECE1-reconstituted (2059)], respectively. *C. albicans* cells were cultured in liquid yeast peptone dextrose (YPD) (Haibo Bio Co., Ltd., HB5193-1, medium containing 10 g/L yeast extract, 20 g/L peptone, and 20 g/L dextrose, corresponding to a 1:2:2 mass ratio), in a shaking incubator (180 rpm) at 30 °C overnight for down-stream experiments. Cultures were washed with sterile PBS (phosphate-buffered saline) and adjusted to the required cell density.

### 2.3 Candidalysin

Synthetic candidalysin (SIIGIIMGILGNIPQVIQIIMSIVKAFK GNK; purity > 95%) was obtained from Shanghai Apeptide Co., Ltd. The peptide was freshly reconstituted in sterile water at 25 mg/mL, aliquoted, and stored at −80 °C until use. All NCM-460 cell experiments described below were performed using synthetic purified candidalysin in the absence of fungal cells. Therefore, these *in vitro* assays evaluated the direct effects of the peptide and did not test a specific *C. albicans* morphotype.

### 2.4 Western blotting

Western blot analyses of NCM-460 cells were performed using [*n* = 3] independent biological experiments, conducted on separate days with independently cultured and treated cells. For the *in vivo* experiments, intestinal tissues from [*n* = 3] individual mice per group were analyzed separately, with each mouse considered one biological replicate. Cells and intestinal tissues were washed with ice-cold PBS and lysed in Western blot lysis buffer (AIWB-012, Qinhe Life Science Co., Ltd.). After incubation on ice for 30 min, the lysates were clarified by centrifugation at 20,000 × *g* for 10 min at 4 °C. Total protein concentrations were determined using a BCA protein-assay kit. Equal amounts of total protein–20 μg per lane for cell lysates and 20 μg per lane for intestinal-tissue lysates–were separated by 10% SDS-PAGE and transferred onto polyvinylidene difluoride membranes. The membranes were blocked with 5% bovine serum albumin in Tris-buffered saline containing 0.1% Tween-20 for 1 h at room temperature and subsequently incubated overnight at 4 °C with primary antibodies against ZO-1 (rabbit, Proteintech, Cat. No. 21773-1-AP, 1:1000), occludin (rabbit, Proteintech, Cat. No. 27260-1-AP, 1:1000), claudin-1 (rabbit, Proteintech, Cat. No. 28674-1-AP, 1:1000), Dsg2 (rabbit, Proteintech, Cat. No. [21880-1-AP], 1:1000), β-actin (rabbit, Proteintech, Cat. No. 20536-1-AP, 1:1000 in NCM-460) and GAPDH (glyceraldehyde-3-phosphate dehydrogenase) (rabbit, Proteintech, Cat. No. 10494-1-AP, 1:1000 in mice tissues). After washing with TBS-T, the membranes were incubated for 1 h at room temperature with HRP-conjugated goat anti-rabbit IgG (Proteintech, Cat. No. [SA00001-2], 1:10000). Protein bands were visualized using an enhanced chemiluminescence substrate and acquired under non-saturating exposure conditions. Band intensities were quantified using ImageJ after local background subtraction. The intensities of ZO-1, occludin, claudin-1, and Dsg2 were normalized to the corresponding β-actin or GAPDH signal from the same lane, and the normalized value of the untreated or uninfected control group was set to 1.0. Dsg2 was included as a desmosomal comparison protein and was not used as a loading control.

### 2.5 Tight junction protein immunofluorescence staining

NCM-460 cells grown on coverslips were treated with vehicle or candidalysin at 3.125, 12.5, or 50 μg/mL for 12 h. These concentrations were selected to represent low-, intermediate-, and high-dose conditions within the dose-response range examined in this study. The concentrations were selected to span a range of epithelial responses under the experimental conditions, from relatively preserved morphology at 3.125 μg/mL to marked TJ-associated changes at 50 μg/mL; 12.5 μg/mL was included as an intermediate condition. A 12-h exposure was selected to permit assessment of junction-associated localization, protein abundance, and transcript responses within a common defined treatment window. After treatment, the cells were gently washed with ice-cold PBS, fixed with freshly prepared 4% paraformaldehyde for 20 min at room temperature. The cells were blocked with 5% bovine serum albumin in PBS for 1 h and then incubated overnight at 4 °C with antibodies against ZO-1 (rabbit, Proteintech, Cat. No. 21773-1-AP), occludin (rabbit, Proteintech, Cat. No. 27260-1-AP), or claudin-1 (rabbit, Proteintech, Cat. No. 28674-1-AP), each diluted 1:500. After washing with PBS containing 0.1% Tween-20, the cells were incubated for 1 h at room temperature in the dark with the appropriate FITC-conjugated goat anti-rabbit IgG secondary antibody (Proteintech, Cat. No. SA00003-2, 1:500). Nuclei were counterstained with DAPI (Beyotime Biotechnology, Cat. No. C1006) for 10 min, and the coverslips were mounted using antifade mounting medium. Images were acquired using an epifluorescence microscope under identical acquisition settings for all groups within each independent experiment. FITC and DAPI signals were detected at excitation/emission wavelengths of approximately 488/525 nm and 358/461 nm, respectively. The analysis included 5 randomly selected fields per condition from *n* = 3 independent biological experiments. Scale bars are shown in all representative images.

### 2.6 FITC-dextran permeability assay

NCM-460 cells were seeded onto 24-well Transwell inserts (PC polycarbonate membrane, 0.4 μm pores, growth area 0.33 cm^2^; Corning) and cultured until a confluent epithelial monolayer had formed (Hammer, 2021). Monolayers were exposed to vehicle or purified candidalysin (3.125, 12.5, or 50 μg/mL) for 12 h. After treatment, the medium in the apical compartment was replaced with serum-free medium containing FITC-dextran (4 kDa; Beyotime, ST2930) at 2000 μg/mL. After 3 h of incubation at 37 °C, 100 μL was collected from the basolateral compartment, and fluorescence was measured using a microplate reader at 482 nm excitation and 520 nm emission. A FITC-dextran standard curve (0–500 μg/mL) was used to verify the fluorescence response. Cell-free inserts containing FITC-dextran were included as barrier-free maximal-flux controls, whereas vehicle-treated NCM-460 monolayers served as the negative control. Three independent biological experiments were performed on separate days, with three technical replicate inserts per condition in each experiment. Technical replicates were averaged to generate one value for each biological replicate. Permeability is presented as lower-chamber FITC-dextran fluorescence normalized to the matched vehicle control. The cell-free insert control was used for assay validation and was excluded from inferential comparisons. Statistical analysis was performed using one-way ANOVA followed by Dunnett’s multiple-comparison test versus vehicle control.

### 2.7 Quantitative real-time PCR (qPCR) analysis of tight junction mRNA expression

To evaluate changes in the mRNA levels of tight junction proteins, NCM-460 human intestinal epithelial cells were treated with varying concentrations of candidalysin for 12 h. Total RNA was extracted from the cells using TRIzol reagent (Thermo Fisher Scientific) according to the manufacturer’s protocol. The concentration and purity of RNA were determined spectrophotometrically using a NanoDrop system (Thermo Fisher Scientific). Approximately 1 μg of total RNA was reverse-transcribed into single-stranded cDNA in a 20 μl reaction system using the Evo M-MLV Reverse Transcription Premix Kit (ACCURATE BIOLOGY AG11728). Quantitative real-time PCR was performed using SYBR Green Pro Taq HS Premix qPCR Reagent (ACCURATE BIOLOGY AG11701) on a real-time PCR system (Bio-Rad). Before qPCR, cDNA was diluted 1:10 (v/v) with nuclease-free water. Two microliters of diluted cDNA, corresponding to 10 ng of input total RNA equivalent, was added to each 20-μL qPCR reaction. The amplification reactions were conducted in triplicate, with each 20 μl reaction mixture containing 10 μl of SYBR Green Master Mix, 0.8 μl of forward primer and 0.8 μl of reverse primer (final primer concentration: 400 nM), 2 μl of diluted cDNA template, and 6.4 μl of nuclease-free water. The thermal cycling conditions were as follows: Initial denaturation at 95 °C for 30 s → 40 cycles of 95 °C for 5 s → 60 °C for 30 s (data acquisition at this step). Primer sequences for target genes (ZO-1, Occludin, Claudin-1) and the reference gene (GAPDH) are listed in Table 1. Relative mRNA expression levels were calculated using the 2^∧^−ΔΔCt method, with normalization to GAPDH, and results are presented as fold changes relative to the untreated control group.

**TABLE 1:** Primer sequences for target genes and the reference gene.

Gene name	Primer sequences (5′–3′)
ZO-1	Forward: AACTGGGCTCTTGGCTTGCTATTC
	Reverse: TCCAGAAGTCAGCACGGTCTCC
Occludin	Forward: AACTTCGCCTGTGGATGA CTTCAG
	Reverse: GACCTTCCTGCTCTTCCCT TTGC
Claudin-1	Forward: GCTGTGGATGTCCTGCGTGTC
	Reverse: GAGGATGCCAACCACCATCAAGG
GAPDH	Forward: GATTCCACCCATGGCAAATTC
	Reverse: CTGGAAGATGGTGATGGGATT

### 2.8 Murine intestinal candidal infection model

Female KM mice (4–6 weeks old, specific-pathogen-free) were randomly assigned to experimental groups, including strain-specific infection groups with various *Candida albicans* strains, a sham-operated control group, and a colon perforation–positive control group. All procedures were approved by the Animal Ethics Committee of Jining Medical University (Protocol No. JNMC-2025-DW-124).

#### 2.8.1 Surgical infection procedure

After 12 h of fasting with free access to water, mice were anesthetized with 2% isoflurane and subjected to a midline laparotomy of approximately 1.5 cm under aseptic conditions. The terminal ileum was gently externalized, and a 34G needle was inserted about 10 cm proximal to the ileocecal junction. A 150 μL suspension of *Candida albicans* (1 × 10^7^ CFU/mL in PBS) or sterile PBS (for the sham-operated group) was slowly injected over 30 s. The puncture site was immediately sealed with surgical bio-glue (Vetbond™ Tissue Adhesive, 3M Company). In the positive control group, colon perforation was induced by puncturing the ascending colon with an 18G needle and extruding feces. This model was selected to provide synchronized exposure to a defined intraluminal fungal inoculum and to facilitate direct comparison of isogenic strains. It was not intended to reproduce the natural sequence of gastrointestinal colonization preceding invasive candidiasis.

#### 2.8.2 Pathogenetic outcome evaluation

Pathogenetic outcomes were assessed in two independent experiments. In the first (*n* = 9 per group), mice were monitored for 72 h to record mortality and clinical signs at 2–4-h intervals. In the second (*n* = 5 per group), conducted 48 h after infection, retro-orbital blood was collected under anesthesia, followed by euthanasia via cervical dislocation. The ileum (injection site), colon, and intestinal contents were harvested for Western blot detection of tight junction proteins, fungal load quantification, hematoxylin and eosin (H&E) staining and periodic acid-Schiff (PAS) staining, and immunohistochemical analysis of tight junction proteins. Serum was separated by centrifugation (3000 × *g*, 10 min, 4 °C) for cytokine analysis, while major organs including the liver, spleen, and kidneys were collected and fixed in 10% neutral-buffered formalin for histopathological examination.

### 2.9 Histopathological and immunohistochemical assessment

Ileal and colonic tissue segments collected at 48 h post-infection was fixed in 10% neutral-buffered formalin for 24 h, processed with graded ethanol, embedded in paraffin, and sectioned into 4 μm-thick slices using a rotary microtome (Leica RM2235).

#### 2.9.1 H&E staining and histopathological scoring

Sections were dewaxed with xylene, rehydrated with graded ethanol, stained with Mayer’s hematoxylin for 5 min and eosin Y for 3 min, then mounted with resin medium. Histopathological scoring included: (1) Epithelial damage (0–1 point: intact or ulcerated); (2) Inflammatory cell infiltration (0–4 points: none to transmural infiltration).

#### 2.9.2 PAS staining

Sections were oxidized with 0.5% periodic acid for 10 min, treated with Schiff’s reagent for 15 min, and then counterstained with hematoxylin. *Candida hyphae*/invasion foci were quantitatively counted per 10 high-power fields (HPF, 100×).

#### 2.9.3 Immunohistochemistry (IHC)

Dewaxed sections were subjected to heat-induced antigen retrieval in citrate buffer (pH 6.0, 95 °C) for 20 min. Endogenous peroxidase was quenched with 3% hydrogen peroxide for 15 min. After blocking with 5% bovine serum albumin for 30 min, the sections were incubated overnight at 4 °C with the following primary antibodies: ZO-1 (rabbit anti-ZO1, 1:200, Proteintech); Occludin (mouse anti-Occludin, 1:200, Proteintech); Claudin-1 (rabbit anti-Claudin-1, 1:200, Proteintech). Subsequently, the sections were incubated with horseradish peroxidase (HRP)-conjugated secondary antibodies (Proteintech) at room temperature for 1 h. Signal detection was performed using 3,3′-diaminobenzidine (DAB) substrate peroxidase substrate, followed by counterstaining with hematoxylin.

### 2.10 Quantification of hematogenous dissemination by droplet digital PCR

Total DNA was isolated from 200 μL of whole blood using the QIAamp DNA Blood Maxi Kit (Qiagen) according to the manufacturer’s protocol. DNA concentrations were quantified via NanoDrop assay. A species-specific 120-bp segment within the *C. albicans* ACT-1 gene, designed for a set of primers: Forward: 5′-TCTGACTCCTGAGGAGAAGTCTGCC-3′; Reverse: 5′-CATGCCCAGTTTCTATTGGTCTCCTT-3′, and Probe: FAM-5′-CCTTGCCCCACAGGGCAGTAACGG-BHQ1-3′ (Integrated DNA Technologies). Amplication set up with reaction mixture containing 1× ddPCR Supermix for Probes (Bio-Rad), 900 nM primers, 250 nM probe, and 50 ng template DNA in 22 μL. Following droplet generation (QX200 Droplet Generator, Bio-Rad), PCR amplification was performed under the following conditions: 95 °C for 10 min; 40 cycles of [94 °C for 30 s → 60 °C for 60 s]; 98 °C for 10 min (ramp rate: 2 °C/s). Quantification and controls: Droplets were analyzed on the QX200 Droplet Reader (Bio-Rad) using QuantaSoft v2.0. Threshold determination utilized a two-component Gaussian mixture model. Each run included: Negative controls, Positive controls (genomic DNA from *C. albicans* SC5314). Limit of detection (LOD): Statistically defined as 3 positive droplets per well. Fungal burden was expressed as genomic equivalents per mL amplitude calculated.

### 2.11 *In vitro* scanning electron microscopy (SEM) analysis

Cells were seeded at a density of 5 × 10^4^ cells/well onto poly-L-lysine-coated coverslips in 24-well plates. When the cell confluence reached 80%, the cultures were treated as follows: experimental group: 50 μg/mL purified candidalysin [with a purity of ≥95% as detected by high-performance liquid chromatography (HPLC)]; Control group: vehicle (phosphate-buffered saline, PBS) (3 biological replicates per group; incubation for 12 h). After treatment, the cells were immediately fixed *in situ* with 2.5% glutaraldehyde in 0.1M sodium cacodylate buffer (pH7.4) at room temperature for 90 min, rinsed 3 times with sodium cacodylate buffer, and treated with 1% osmium tetroxide (OsO_4_) in the same buffer for 90 min, followed by gradient dehydration with ethanol (30%, 50%, 70%, 90%, 100%; each concentration for 10 min). The dried samples were mounted on aluminum stubs and sputter-coated with 10-nm gold-palladium under an argon atmosphere. Imaging was performed using a JEOL JSM-7900F scanning electron microscope. Ten randomized fields per coverslip were captured at magnifications ranging from 1,000× to 10,000×.

### 2.12 Statistical analysis

The significance level was determined by one-way analysis of variance (ANOVA) combined with Dunnett’s multiple comparison correction. The difference was considered statistically significant when *P* < 0.05. The experiment was performed with at least three independent biological replicates. No formal *a priori* power calculation was performed; sample sizes were selected on the basis of the exploratory design, feasibility, and animal-welfare considerations. Data were analyzed using GraphPad Prism 8.0.

## 3 Results

### 3.1 Genetic deletion of ECE1 ameliorates intestinal pathogenesis in mice

The cytolytic peptide toxin candidalysin, encoded by the ECE1 gene in *Candida albicans*, plays a critical role in intestinal invasion and pathogenesis. To assess its contribution, we employed isogenic strains, including the wild type [WT (1477)], ECE1 knockout [ece1Δ/Δ (2057)], and ECE1-reconstituted (2059), in a murine infection model.

At 48 h post-infection, histopathological analyses revealed that intestinal inflammation was strongly dependent on *ECE1* expression. Mice infected with the WT (1477) strain developed severe intestinal inflammation, with marked disruption of the gastrointestinal lining. Hematoxylin and eosin (HE) staining showed complete loss of mucosal architecture, including folds, villi, and crypts (Figure 1A), and histopathological inflammatory scores were significantly higher than in negative controls (Figure 1B, *p* < 0.001). Infection with the ECE1-reconstituted (2059) strain produced similarly extensive inflammation, with a mean injury score of 4, not significantly different from that observed in the WT (1477) group (*p* > 0.05). In contrast, mice infected with the ece1Δ/Δ (2057) strain exhibited markedly reduced intestinal inflammation, preservation of mucosal structures, and significantly lower injury scores compared with both the WT and ECE1-reconstituted groups (Figure 1B). Representative histological sections further confirmed extensive mucosal ulceration, neutrophil infiltration, and edema in the WT (1477)- and ECE1-reconstituted (2059)-infected groups, whereas tissues from the ece1Δ/Δ (2057) group displayed only minor, focal inflammatory changes with limited immune cell infiltration (Figure 1A).

**FIGURE 1:**
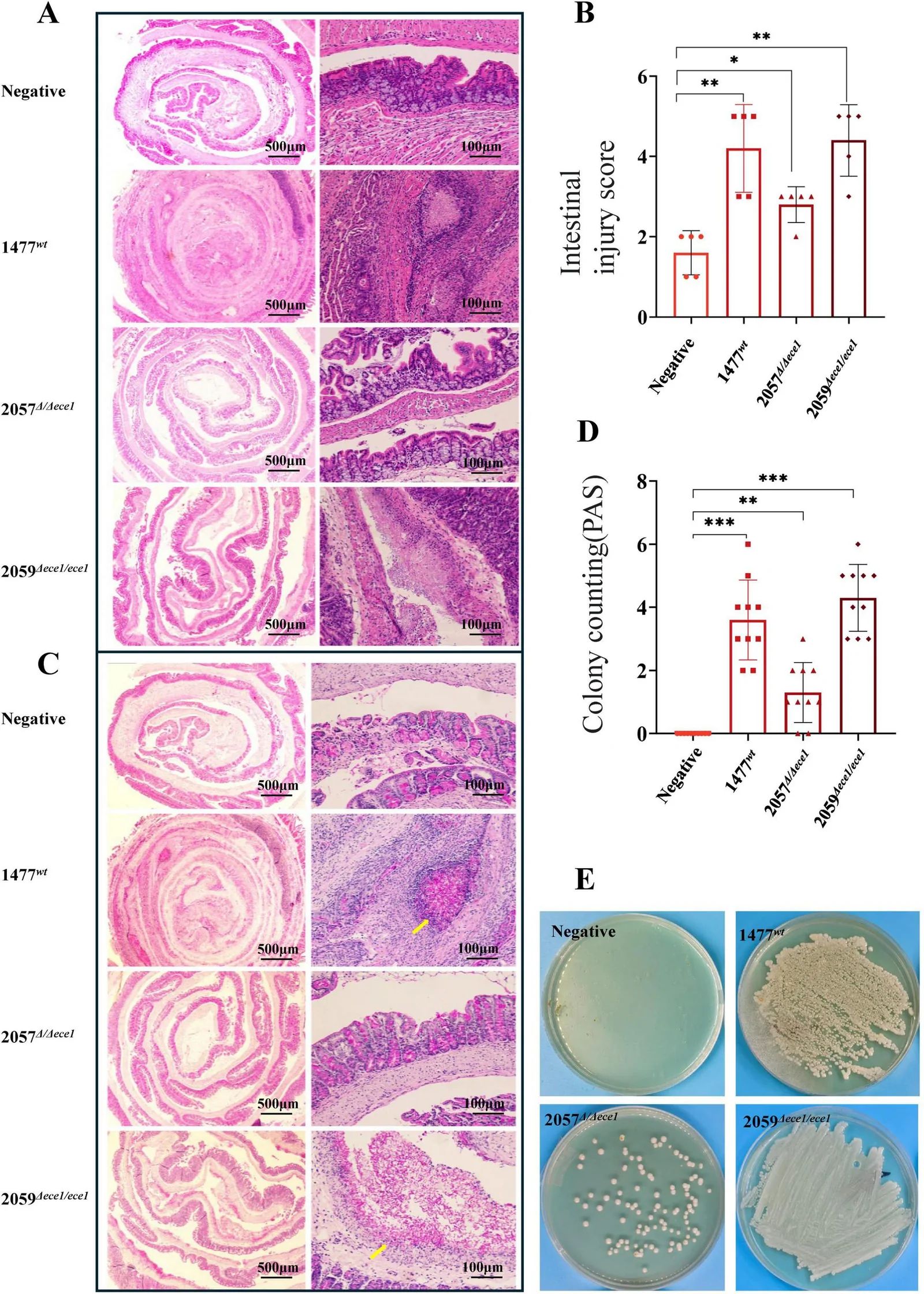
Intestinal pathology and tissue/fecal fungal burden after infection with isogenic *C. albicans* strains. **(A)** Representative H&E-stained ileal sections harvested 48 h after inoculation from sham-operated controls, WT (1477)-infected mice, ece1Δ/Δ (2057)-infected mice, and ECE1-reconstituted (2059)-infected mice. Scale bars, 500 μm (low magnification) and 100 μm (high magnification). **(B)** Histopathology scores for the sections shown in panel **(A)** . **(C)** PAS-stained sections; arrows indicate representative PAS-positive fungal structures. Scale bars, 500 μm and 100 μm. **(D)** Quantification of PAS-positive fungal structures per 10 high-power fields. **(E)** Representative fungal-recovery plates from fecal samples collected at 48 h after infection, with quantitative CFU shown in the accompanying analysis. The faint red signal in negative-control sections was considered nonspecific background and was not counted as fungal staining. Data are shown for *n* = 5 mice per group. *p < 0.05; **p < 0.01; ***p < 0.001; ns, not significant.

These pathological findings correlated with fungal invasion and burden. Periodic acid–Schiff (PAS) staining revealed abundant fungal cells in the intestinal mucosa of mice infected with WT (1477) or ECE1-reconstituted (2059) strains, with no significant difference between the two groups (Figures 1C, D). Quantitative analysis of fecal fungal burden likewise showed similarly high recovery levels in both groups at 48 h post infection (*p* < 0.001 versus control; Figure 1E). By contrast, infection with the ece1Δ/Δ (2057) strain resulted in significantly reduced tissue-associated *C. albicans* (Figures 1C, D). Fecal CFU counts were also markedly lower in the ece1Δ/Δ group than in the WT and ECE1-reconstituted groups (*p* < 0.01) as early as 48 h post-infection (Figure 1E). Consistent with the PAS staining and reduced mucosal injury, the pronounced reduction in fecal CFU in the absence of *ECE1* demonstrated a markedly impaired ability to colonize and damage the gastrointestinal tract. In contrast, the WT (1477) and ECE1-reconstituted (2059) groups maintained high fungal populations in intestinal site, accompanied by more severe mucosal damage and inflammation. Together, these findings indicate that ECE1/candidalysin contributes to intestinal fungal persistence/recovery, mucosal injury, and inflammation in this controlled infection model. They do not establish that candidalysin is indispensable for gastrointestinal colonization under natural conditions.

### 3.2 Candidalysin deficiency attenuates systemic organ damage and mortality following intestinal infection

In line with the localized intestinal pathology and colonization deficits, loss of ECE1 profoundly reduced systemic disease following intestinal infection. Histopathological analysis of vital organs at 48 h revealed striking strain-dependent differences (Figure 2). Mice infected with the WT (1477) or ECE1-reconstituted (2059) strains developed severe invasive candidiasis, with characteristic organ-specific damage. These included myocarditis with mononuclear infiltration and focal necrosis in the heart, multifocal inflammatory lesions with hepatocyte degeneration and microabscess formation in the liver, marked lymphoid depletion and extramedullary hematopoiesis in the spleen, tubulointerstitial nephritis with epithelial injury in the kidneys, and diffuse interstitial pneumonitis with alveolar edema and hemorrhage in the lungs. In contrast, infection with the ece1Δ/Δ (2057) strain resulted in only minimal inflammatory infiltrates or focal damage, and overall organ architecture remained largely preserved. Together, these findings indicate that ECE1-encoded candidalysin contributes to local intestinal pathogenesis, systemic dissemination, and organ injury in this intestinal-derived candidiasis model.

**FIGURE 2:**
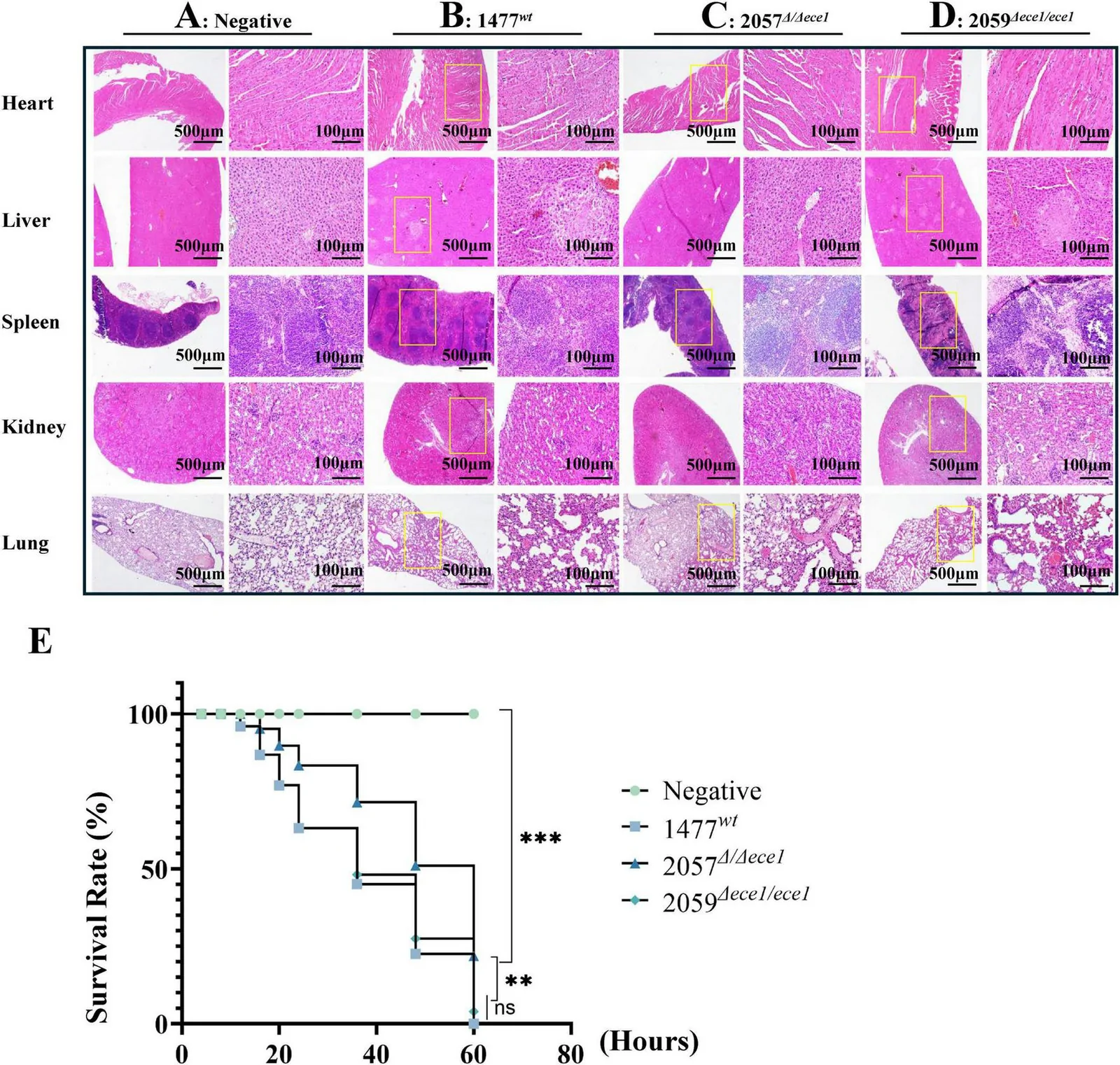
Histopathological changes in the terminal phenotyping cohort and survival in an independent cohort after intestinal infection. **(A)** Sham-operated control tissues. **(B)** WT (1477)-infected tissues. **(C)** ece1Δ/Δ (2057)-infected tissues. **(D)** ECE1-reconstituted (2059)-infected tissues. Representative H&E-stained sections of heart, liver, spleen, kidney, and lung were collected 48 h after infection from the terminal phenotyping cohort. Boxes/arrows identify representative inflammatory infiltration, necrosis, epithelial injury, or structural disruption. Verified scale bars are indicated in each panel. **(E)** Kaplan–Meier survival curves generated from a separate survival cohort monitored for 72 h. Survival data are shown for 9 mice per group and were analyzed using the log-rank (Mantel–Cox) test. The terminal phenotyping cohort included *n* = 5 mice per group, and the survival cohort included *n* = 9 independent mice per group. **p < 0.01; ***p < 0.001.

These pathological differences translated directly into survival outcomes (Figure 2E). By day 3 post-infection, uninfected control mice showed no mortality, but all WT (1477)-infected mice had succumbed, and mortality in the ECE1-reconstituted (2059) group also reached 100%. Strikingly, although a significant improvement compared with candidalysin-producing strains (*p* < 0.01), 75% of ece1Δ/Δ (2057)-infected mice did not survive to the endpoint. Together, these results indicate that ECE1/candidalysin is an important contributor to systemic dissemination, multi-organ injury, and mortality in this model, but the residual mortality in ece1-mutant-infected mice indicates that candidalysin-independent fungal and host mechanisms also contribute substantially to disease severity.

### 3.3 Candidalysin facilitates hematogenous dissemination in the intestinal infection model

To investigate how *C. albicans* disseminates from the gastrointestinal tract to the bloodstream and damages distant organs, and to determine the contribution of candidalysin to this process, we next quantified fungal genomic DNA in peripheral blood using droplet digital PCR (ddPCR). Primers were designed to target a *C. albicans*–specific genomic sequence, allowing sensitive detection of circulating fungal DNA.

Consistent with the patterns of multi-organ pathology, candidemia varied significantly among the infection groups (Figure 3). Positive droplet PCR for *Candida* genomic DNA signals were detected in 57% (4 of 7) of mice infected with candidalysin-producing strains [WT (1477) and ECE1-reconstituted (2059)]. At 48 h post-infection, the mean positive-droplet amplitude in WT (1477)-infected mice was 3,236 (range from 2,218 to 4,431), while mice infected with the ECE1-reconstituted (2059) strain showed a comparable mean positive-droplet amplitude of 2,100 (range from 1,930 to 2,305). In contrast, only 14% (1 of 7) of mutant-infected mice produced positive ddPCR signals above the detection threshold, and this ddPCR positive mouse infected with the ece1Δ/Δ (2057) strain exhibited a substantially lower positive-droplet amplitude (810) than the WT and ECE1-reconstituted groups. These analyses support the conclusion that ECE1-mediated mucosal injury contributes to or facilitates bloodstream invasion. The low-level positive signal in one ece1Δ/Δ-infected mouse indicates that dissemination can still occur in the absence of ECE1.

**FIGURE 3:**
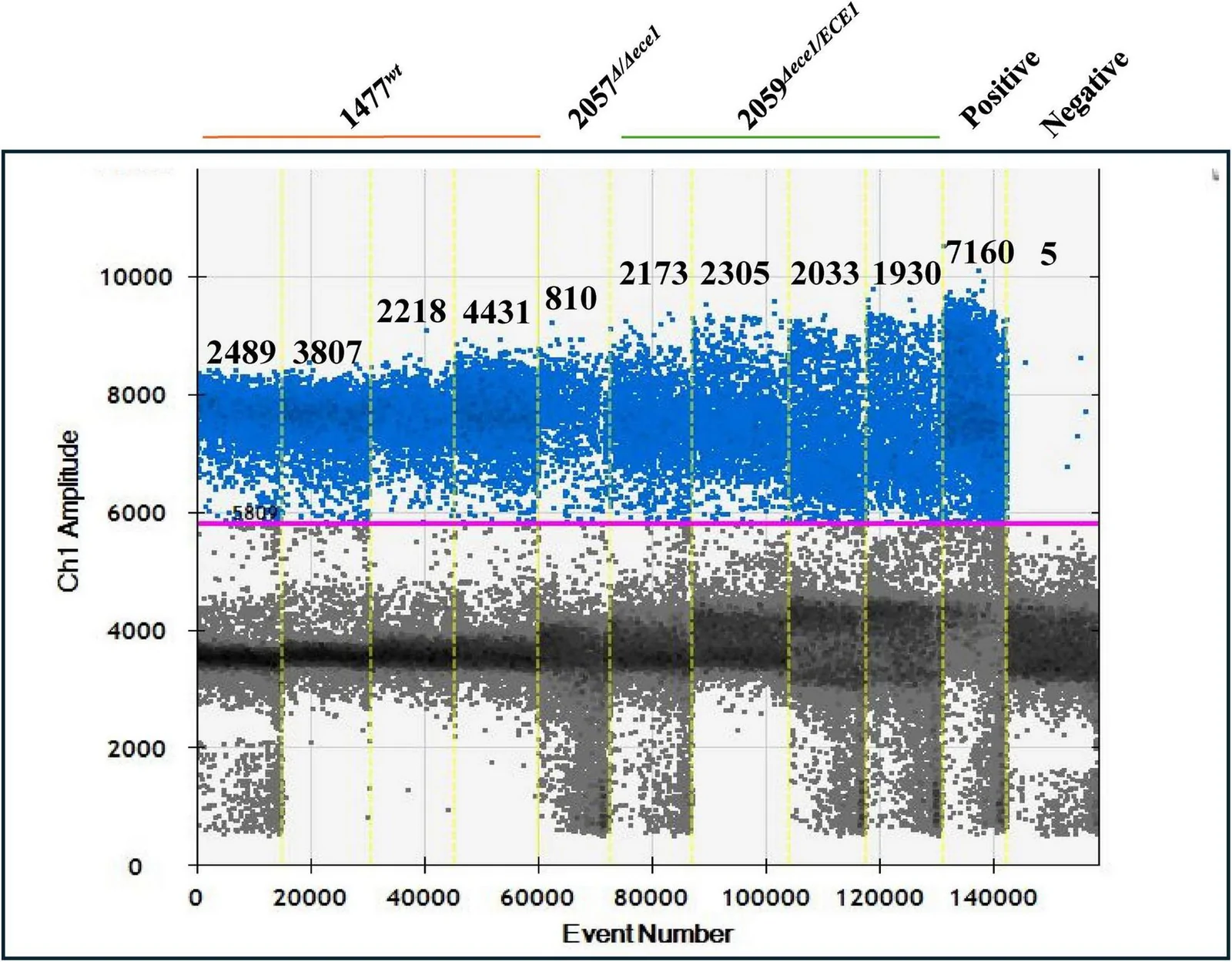
Candidalysin promotes efficient hematogenous dissemination of *C. albicans*. Droplet digital PCR (ddPCR) quantification of *C. albicans* genomic DNA in peripheral blood collected 48 h post-infection. Primers targeting a *C. albicans* ACT-1 sequence were used to assess fungal DNA loads in peripheral blood from mice infected withWT (1477), ece1Δ/Δ (2057), or ECE1-reconstituted (2059) strains. Positive ddPCR signals (+) were achieved with 50 ng genomic DNA extracted from a *C. albicans* SC5314 culture, whereas negative signals (−) represent samples without DNA. Data are shown as positive-droplet amplitude values for each individual blood sample; *n* = 7 mice per group.

### 3.4 Intestinal-derived candidiasis promotes systemic inflammation

Although candidalysin plays an important role in fungal dissemination into the bloodstream (Figure 3), animals infected with the ECE1 mutant nevertheless still succumbed to infection (Figure 2E). This discrepancy suggests that candidalysin-deficient strains may still provoke lethal host responses through mechanisms other than sustained fungemia, such as cytokine-driven systemic inflammation. To test this, we quantified serum inflammatory cytokines by ELISA.

As shown in Figures 4A–C, mice infected with the WT (1477) strain developed marked systemic cytokine responses, with markedly elevated levels of TNF-α (987.7 ± 328.2 pg/mL), IL-1β (125.4 ± 3.0 pg/mL), and IL-6 (76.8 ± 32.6 pg/mL). Comparable cytokine elevations were observed in mice infected with the ECE1-reconstituted (2059) strain (TNF-α: 1006.4 ± 455.8 pg/mL, IL-1β: 133.9 ± 12.6 pg/mL, and IL-6: 58.2 ± 10.2 pg/mL). Interestingly, infection with the ece1Δ/Δ (2057) strain resulted in IL-6 levels comparable to the reconstituted strain (56.7 ± 18.1 pg/mL) and a modest reduction in IL-1β (113.0 ± 13.7 pg/mL; *p* < 0.05). By contrast, TNF-α was nearly halved (522.7 ± 189.0 pg/mL) compared with WT and reconstituted strains (*p* < 0.01).

**FIGURE 4:**
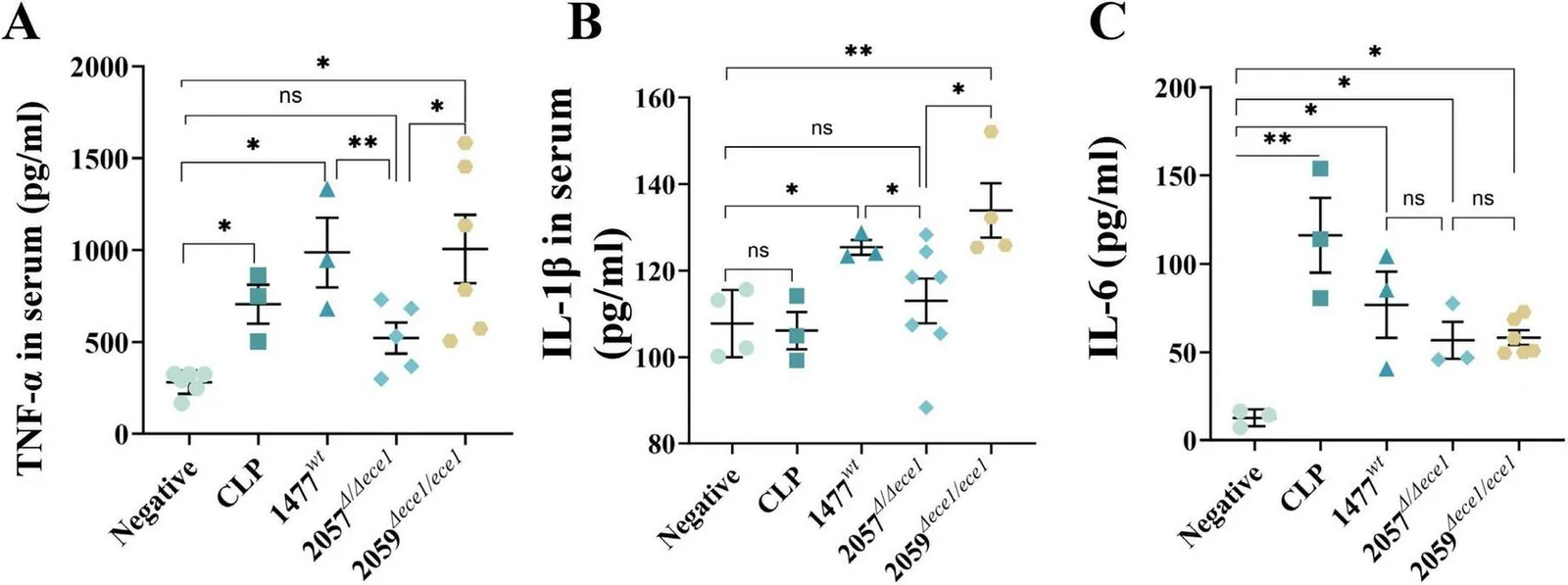
Serum cytokine responses 2 days after infection with *C. albicans* WT and mutant strains. **(A–C)** Levels of pro-inflammatory cytokines TNF-α **(A)**, IL-1β **(B)**, and IL-6 **(C)** were quantified by ELISA in sera from control mice and mice infected with three isogenic strains. Data are presented as mean ± SEM, with statistical significance indicated. A surgical colon-perforation control without *Candida* inoculation was included to distinguish responses related to mechanical intestinal injury from infection-associated responses. *p < 0.05; **p < 0.01; ns, not significant.

To isolate cytokine responses arising from mucosal injury independent of *Candida* or candidalysin, colon perforation (CLP) was used as a comparator (Figure 4). CLP significantly increased IL-6 and TNF-α (*p* < 0.01 and *p* < 0.05, respectively), whereas IL-1β remained unchanged, in contrast to its robust induction during WT infection. These findings highlight IL-1β as a candidiasis-specific response, while IL-6 and TNF-α can be driven by both mechanical injury and infection. Importantly, the marked reduction of TNF-α in mutant-infected mice is consistent with a candidalysin-associated contribution to the TNF-α response that contributes to mucosal injury. The residual systemic response observed in ECE1-deficient infections–characterized by sustained IL-1β and IL-6–may contribute to the persistent mortality, although causality was not tested despite lower fungal burden and reduced pathology.

### 3.5 Candidalysin disrupts intestinal tight junction proteins

To determine the effect of candidalysin on intestinal epithelial barrier integrity during *Candida* dissemination, we examined the expression of core tight junction proteins (ZO-1, Occludin, and Claudin-1) using Western blot (WB) and immunohistochemistry (IHC). At 48 h post-infection, WB analysis revealed that the protein levels of ZO-1, Occludin, and Claudin-1 were markedly reduced in intestinal tissues from mice infected with WT (1477) and ECE1-reconstituted (2059) strains compared with uninfected controls, consistent with marked TJ-associated molecular and structural injury (Figure 5A). In contrast, mice infected with the ece1Δ/Δ (2057) strain showed only slight reductions in these proteins, with expression largely preserved, supporting an association between candidalysin expression and the loss of these TJ-associated proteins.

**FIGURE 5:**
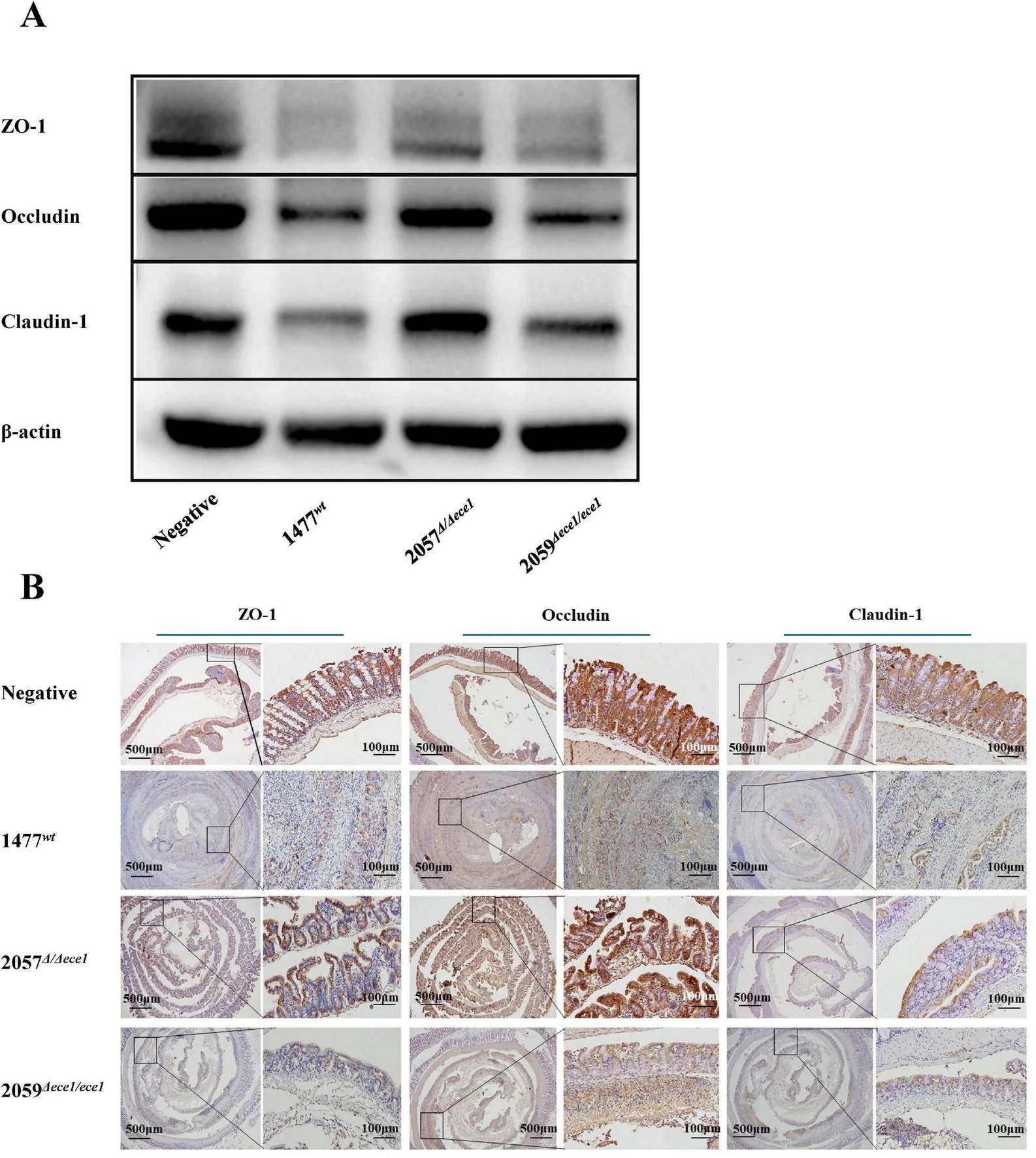
Candidalysin disrupts intestinal tight junction proteins. **(A)** Western blot analysis of intestinal tissues at 48 h post-infection showing expression levels of tight junction proteins ZO-1, Occludin, and Claudin-1. Protein levels were reduced in tissues from mice infected with WT and ECE1-reconstituted strains, whereas expression in ece1Δ/Δ-infected mice was relatively preserved. **(B)** Immunohistochemical staining of intestinal mucosa for ZO-1, Occludin, and Claudin-1. Loss of protein expression and disrupted mucosal structure were observed in WT (1477) and ECE1-reconstituted (2059) infections, whereas continuous and well-distributed staining was maintained in ece1Δ/Δ (2057)-infected tissues, similar to negative control mice. The staining pattern was consistent with strain-dependent TJ disruption during intestinal infection.

Consistent with WB results, IHC analysis demonstrated substantial loss of ZO-1, Occludin, and Claudin-1 in the intestinal mucosa of WT (1477) and ECE1-reconstituted (2059)-infected mice, accompanied by weakened staining intensity and disrupted mucosal architecture (Figure 5B). Conversely, ece1Δ/Δ (2057)-infected mice exhibited continuous and evenly distributed staining of these proteins, particularly ZO-1 and Occludin, comparable to uninfected controls. Together, these findings are consistent with an association between candidalysin expression and intestinal TJ disruption.

### 3.6 Concentration-dependent effects of candidalysin on TJ-associated signals in NCM-460 cells

To validate the crucial effects of ECE1/candidalysin *in vivo* mucosal system, we assessed the effects of purified candidalysin on tight junction (TJ) integrity using the NCM-460 colonic epithelial cell line. In this *in vitro* model, we evaluated the dose-dependent response of core TJ proteins, including ZO-1, Occludin, and Claudin-1, under varying concentrations of candidalysin (Figure 6).

**FIGURE 6:**
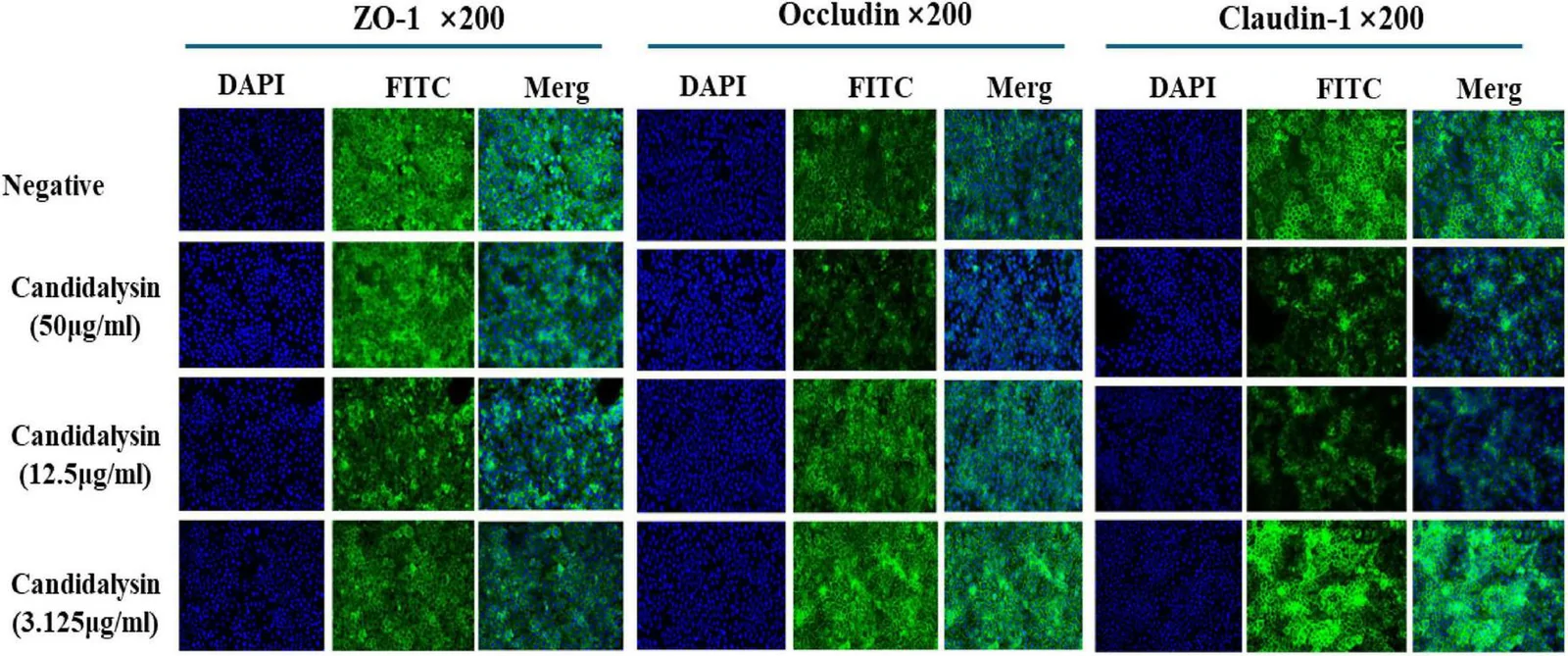
Biphasic modulation of tight junction proteins by candidalysin in NCM-460 colonic epithelial cells. Immunofluorescence analysis of ZO-1, Occludin, and Claudin-1 (200× magnification) following exposure of intestinal epithelial cells to different concentrations of candidalysin. Representative immunofluorescence images are shown for vehicle and candidalysin at the indicated concentrations. At 3.125 μg/mL, junction-associated signals were relatively preserved. At 12.5 and 50 μg/mL, junction-associated signals were reduced and showed altered localization, with Occludin and Claudin-1 particularly affected. Primary antibodies against each individual TJ protein were used at 1:500 dilution, followed by FITC-conjugated goat anti-rabbit secondary antibodies for detection. Nuclei were counterstained with DAPI.

Immunofluorescence microscopy revealed a biphasic effect of candidalysin on TJ proteins. At low concentrations (3.125 μg/mL), TJ continuity was preserved, and fluorescence intensity of Occludin and Claudin-1 was modest but consistently increased compared with unstimulated controls, suggesting relative preservation of junction-associated signals at this concentration (Figure 6). In contrast, intermediate concentrations (≥12.5 μg/mL) induced pronounced structural disintegration, characterized by cytoplasmic mislocalization and significant loss of fluorescence signals for all three TJ proteins (Figure 6). Exposure to high concentrations (50 μg/mL) led to near-complete dissolution of tight junctions, with Occludin and Claudin-1 particularly affected (Figure 6).

The increased FITC-dextran flux observed at 12.5 and 50 μg/mL (Supplementary Figure 1) was consistent with impaired paracellular barrier function under these conditions. At the lowest tested concentration (3.125 μg/mL), the mixed protein and transcript response, together with the absence of increased FITC-dextran flux, did not establish barrier reinforcement.

### 3.7 FITC-dextran flux supports increased epithelial permeability at higher candidalysin concentrations

To determine whether candidalysin-associated changes in TJ-associated proteins were accompanied by functional alterations in epithelial barrier permeability, FITC-dextran flux across NCM-460 monolayers was quantified in 24-well Transwell inserts. FITC fluorescence increased with increasing FITC-dextran concentration in the standard curve (R^2^ = 0.995; Supplementary Figure 1). Relative to vehicle-treated monolayers, exposure to 3.125 μg/mL candidalysin did not increase FITC-dextran flux (0.92-fold of vehicle; *P* = 0.584). In contrast, FITC-dextran flux increased to 2.14-fold and 4.00-fold of vehicle after exposure to 12.5 and 50 μg/mL candidalysin, respectively (both *P* < 0.0001; Supplementary Figure 1). Cell-free inserts showed high tracer flux, supporting the dynamic range of the assay. These data provide functional support for increased epithelial permeability at higher candidalysin concentrations, while the low-dose condition did not show increased permeability.

### 3.8 Candidalysin actively represses transcription of tight junction genes

To confirm the dose mechanisms underlying candidalysin-mediated epithelial responses observed in Figure 6, we examined both protein and transcriptional regulation of core tight junction (TJ) proteins in NCM-460 cells exposed to low (3.125 μg/mL) and high (50 μg/mL) concentrations of candidalysin, with desmoglein-2 (Dsg2) included as a desmosomal comparison protein.

Western blot analysis demonstrated variable concentration-dependent effects of candidalysin among three tight junction proteins (Figures 7A–E). Unlike the largely unchanged ZO-1 fluorescence signal at 3.125 μg/mL (Figure 6), ZO-1 protein abundance was significantly higher than the vehicle control at this concentration (*p* < 0.05) and was markedly reduced at 50 μg/mL (*p* < 0.01; Figure 7B). Despite the enhanced fluorescent signals, Occludin expression remained largely unchanged across both concentrations (Figure 7D) when compared to control. Claudin-1 levels were significantly decreased at 50 μg/mL (*p* < 0.01) and slightly decreased at 3.125 μg/mL (*p* < 0.05 versus control) (Figure 7E). Meanwhile, Dsg2 expression was stable under candidalysin-stimulated and unstimulated conditions, confirming assay reliability (Figure 7C). Scanning electron microscopy (SEM) further showed high-dose candidalysin-associated structural disruption. As shown in Figure 7F, compared with the intact polygonal morphology with well-defined junctions in untreated cells, exposure to 50 μg/mL led to marked structural collapse, with loss of cell contours and marked disorganization of junctional elements.

**FIGURE 7:**
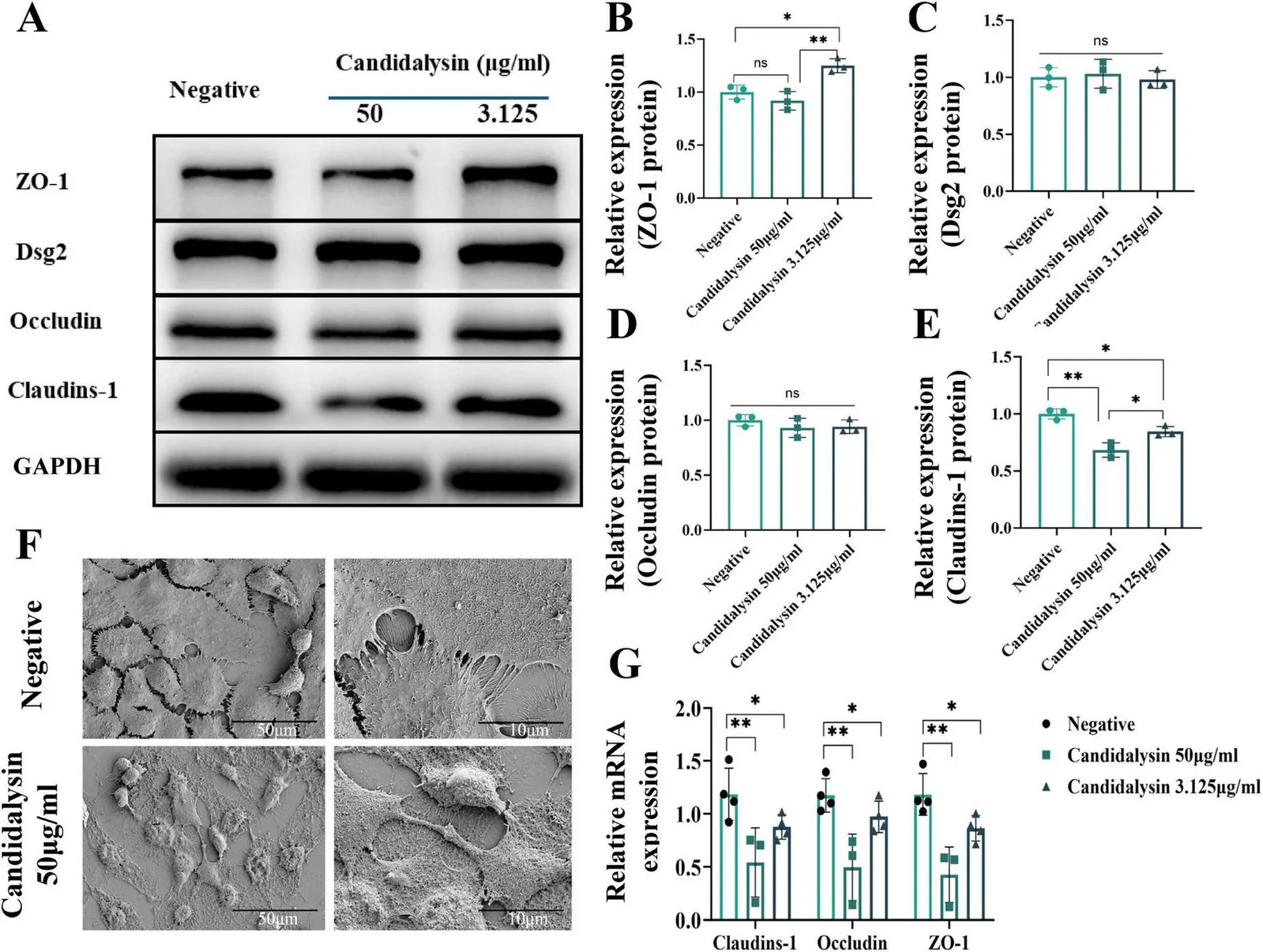
Candidalysin regulates tight junction proteins at both protein and transcript levels in colonic epithelial cells. **(A)** Representative Western blot analysis of ZO-1, Occludin, Claudin-1, and desmoglein-2 (Dsg2) in NCM-460 cells treated with 3.125 μg/mL or 50 μg/mL candidalysin. GAPDH was used as the loading control, whereas Dsg2 was included as a desmosomal comparison protein. **(B–E)** Densitometric quantification of TJ proteins from *n* = 3 independent biological experiments. ZO-1 expression was significantly increased at 3.125 μg/mL but markedly reduced at 50 μg/mL. Claudin-1 showed significant reduction at 50 μg/mL, while Occludin levels remained unchanged at both concentrations. **(F)** Scanning electron microscopy images showing intact polygonal morphology with preserved junctions in untreated cells versus marked structural collapse of cell structures and junctional disintegration after 50 μg/mL candidalysin exposure. **(G)** qPCR analysis of ZO-1, Occludin, and Claudin-1 transcripts showing significant downregulation at both low and high candidalysin concentrations, with greatest reduction observed at 50 μg/mL.*p < 0.05; **p < 0.01.

To determine whether transcript-level changes accompanied the observed protein changes, we analyzed transcriptional regulation of three TJ genes. Surprisingly, qPCR analysis revealed significant downregulation of ZO-1, Occludin, and Claudin-1 transcripts at the high toxin concentration (50 μg/mL) (*p* < 0.01) (Figure 7G). A slight downregulation of all three genes was also observed at 3.125 μg/mL (*p* < 0.05 vs. control). The accompanying transcript reductions suggest that altered gene expression may contribute to the TJ-associated phenotype. However, the present study does not distinguish transcriptional regulation from changes in protein turnover, cell injury, or other post-translational mechanisms.

## 4 Discussion

This study identifies candidalysin as a major contributor to intestinal epithelial tight-junction (TJ) disruption and to gut-to-blood dissemination of *Candida albicans* in a controlled intraluminal infection model, while at the same time showing that it is not the sole determinant of invasive disease. The central significance of these findings is that the epithelial barrier–specifically the organization and abundance of TJ components such as ZO-1, occludin, and claudin-1 –represents a therapeutically tractable node at which candidalysin acts to open a route for fungal translocation. Because barrier loss precedes bloodstream invasion, interventions that neutralize candidalysin or preserve junctional integrity may narrow the window for dissemination in susceptible hosts.

Prior work has established that *C. albicans* can traverse intestinal epithelial barriers and that candidalysin mediates a substantial component of the associated epithelial damage, although low-level toxin-independent translocation may still occur. A foundational study by Allert et al. (2018) demonstrated that candidalysin-dependent epithelial injury is a major determinant of fungal translocation across intestinal barriers. More recently, a gut-on-chip study by Morelli and Queiroz (2025) showed that candidalysin impairs epithelial integrity and induces inflammatory responses in a human intestinal epithelial model. Building on these important studies, our work provides complementary *in vivo* genetic evidence linking candidalysin status to mucosal injury, circulating fungal DNA signals, and distal-organ pathology. Nevertheless, our results do not establish candidalysin as the sole driver of invasive disease, nor do they demonstrate direct biochemical cleavage of TJ proteins. Candidalysin activity is likely to be context dependent, as its dynamic expression has been associated with both commensal-like colonization and pathogenic behavior *in vivo* (Fróis-Martins et al., 2025; Liang et al., 2024).

At the cellular level, exposure to high concentrations of candidalysin was associated with reduced junctional staining and lower transcript abundance of selected TJ components, and this molecular phenotype coincided with increased paracellular permeability measured by FITC-dextran flux. These observations provide functional support for epithelial barrier compromise under high-toxin conditions. However, the present experiments cannot distinguish whether the reduced TJ transcript and protein signals resulted from transcriptional repression, altered protein turnover, generalized epithelial injury, changes in cell number, or other post-translational mechanisms. Interpretation of the low-dose response also requires caution. At 3.125 μg/mL, TJ gene transcripts were reduced despite partial preservation or modest increases in selected protein or fluorescence readouts, whereas FITC-dextran permeability was not increased. Thus, this mixed response does not support the conclusion that low-concentration candidalysin reinforces or protects epithelial barrier function. The transcript findings may initially appear counterintuitive because an injured epithelium might be expected to increase junctional-gene expression as a compensatory repair response. We propose that the observed pattern may reflect a mismatch among response timing, molecular compartment, and injury severity. At low concentration, pre-existing junctional proteins may remain stable or redistribute while steady-state transcripts decline. At high concentration, membrane permeabilization, calcium disturbance, inflammatory or stress signaling, and reduced epithelial fitness may exceed the capacity for repair, causing transcript loss, protein loss, and increased permeability. A compensatory response may occur outside the single 12-h sampling point. Because we did not measure nascent transcription, RNA stability, a time course, or parallel viability, these mechanisms remain hypotheses. Future time-course experiments combining transcript measurements, protein turnover, cell viability, and functional barrier assays will be required to distinguish among these possibilities.

Paracellular barrier function depends on the coordinated remodeling of transmembrane TJ components, cytoplasmic scaffolds, and intercellular adhesion complexes rather than on any single junction protein (Horowitz et al., 2023; Kuno et al., 2025; Nur Husna et al., 2021; Raya-Sandino et al., 2023; Zimmer and Kowalczyk, 2024). The largely unchanged desmoglein-2 (Dsg2) signal in our experiments indicates that candidalysin-associated effects were heterogeneous across junction-associated structures; however, this observation does not establish pathway-specific targeting. Because ZO-1 abundance and junctional organization can be influenced by protein turnover and actomyosin-associated mechanisms (Cho et al., 2022; Zhang et al., 2025), the reductions in TJ-associated protein levels observed here should not be interpreted as evidence of direct proteolytic degradation. Similar barrier-disruptive patterns have been described for other pore-forming microbial toxins, suggesting convergence at the level of epithelial injury rather than demonstrating a unique candidalysin-specific mechanism (Liu et al., 2024). Related EGFR-, MAPK/ERK-, and NF-κB-associated pathways have been implicated in epithelial or host responses to *C. albicans* virulence factors and candidalysin (Song et al., 2022; Sprague et al., 2024; Swidergall et al., 2021). However, the present study was not designed to dissect these upstream signaling cascades, and the relative contribution of each pathway to the observed TJ changes remains unresolved.

Our *in vivo* comparisons reinforce the association between ECE1/candidalysin status and disease severity using genetically matched strains to minimize background variation. Loss of ECE1 was associated with reduced intestinal injury, lower circulating fungal-DNA signals, and less distant-organ pathology, whereas ECE1 reconstitution largely restored a WT-like phenotype. These findings support candidalysin as a major contributor to epithelial injury and dissemination in this experimental system. Importantly, even with markedly reduced translocation-associated signals and tissue damage, substantial mortality persisted in the *ece1Δ/Δ* group, together with sustained elevation of IL-1β and IL-6. These findings emphasize that candidalysin amplifies, but does not fully determine, lethal infection outcomes. Residual disease may reflect procedure-related mucosal injury, candidalysin-independent fungal virulence mechanisms, low-level toxin-independent translocation, or host-derived inflammatory dysregulation.

From a translational perspective, neutralizing candidalysin activity or preserving epithelial barrier integrity might limit gut-origin fungal spread in susceptible hosts. Previous studies demonstrating candidalysin-associated epithelial damage and barrier dysfunction provide a rationale for evaluating toxin-targeted and host-directed barrier-protective strategies (Allert et al., 2018; Morelli and Queiroz, 2025). However, therapeutic efficacy cannot be inferred solely from the correlative data presented here. Future studies should distinguish among fungal burden, epithelial viability, inflammatory signaling, and functional barrier rescue, ideally in models that more closely recapitulate natural gastrointestinal colonization preceding invasive candidiasis.

This work has several limitations. First, the surgical intraluminal inoculation model enables synchronized fungal challenge but does not recapitulate the natural sequence of gut dysbiosis, mucosal colonization, and progressive invasive candidiasis. Therefore, the present model should be regarded as a controlled exposure and dissemination model rather than a complete representation of natural colonization-to-invasion pathogenesis. Second, we did not define the signaling pathways driving candidalysin-associated TJ alterations. Although EGFR-, MAPK/ERK-, and NF-κB-related pathways have been implicated in responses to *C. albicans* virulence factors and candidalysin (Song et al., 2022; Sprague et al., 2024; Swidergall et al., 2021), their direct involvement in the present TJ phenotype remains to be established. Finally, the basis of residual mortality in *ece1Δ/Δ* infections remains uncharacterized. Future studies employing targeted pathway perturbation, parallel assessment of cell viability and barrier function, and physiologically relevant gastrointestinal colonization models will be required to define how candidalysin, additional fungal virulence factors, and host responses jointly promote intestinal dissemination *in vivo*.

## 5 Conclusion

Herein, we show that candidalysin is an important contributor to intestinal epithelial TJ disruption and gut-to-blood dissemination in a controlled intestinal infection model. At the low tested concentration, selected TJ-associated protein signals were preserved or modestly increased; however, the mixed protein and transcript findings, together with the absence of increased FITC-dextran flux, do not establish barrier reinforcement. In contrast, higher candidalysin concentrations were associated with reduced ZO-1, occludin, and claudin-1 expression, altered junctional localization, and increased FITC-dextran permeability, supporting a functional contribution of candidalysin to epithelial barrier impairment. These data identify candidalysin-associated epithelial injury and barrier dysfunction as therapeutically relevant features of intestinal candidiasis and provide a rationale for evaluating candidalysin-targeted or barrier-protective strategies in high-risk hosts.

## Data Availability

The original contributions presented in this study are included in this article/Supplementary material, further inquiries can be directed to the corresponding author.

## Supplementary material

Supplementary Figure 1 FITC-dextran flux across NCM-460 monolayers after candidalysin exposure. **(A)** FITC-dextran standard curve (0–500 μg/mL) used to verify the fluorescence response (linear fit, R^2^ = 0.995). **(B)** NCM-460 monolayers grown in 24-well Transwell inserts were treated with vehicle or candidalysin (3.125, 12.5, or 50 μg/mL) for 12 h, followed by measurement of FITC-dextran in the basolateral compartment. Fluorescence values were normalized to the vehicle control. Each filled circle represents one independent biological experiment and is the mean of three technical replicate inserts; horizontal bars indicate mean ± SEM (*n* = 3 independent biological experiments per cell-containing group). Open circles indicate cell-free inserts used as barrier-free maximal-flux controls; these controls were not included in inferential comparisons. One-way ANOVA with Dunnett’s multiple-comparison test versus vehicle control was used. *****p* < 0.0001; ns, not significant.
